# Using patient and public involvement to improve the research design and funding application for a project aimed at fostering a more collaborative approach to the NHS health check: the CaVIAR project (better Care Via Improved Access to Records)

**DOI:** 10.1186/s40900-018-0101-7

**Published:** 2018-06-11

**Authors:** Brian McMillan, Sarah Fox, Moira Lyons, Suzy Bourke, Manoj Mistry, Angela Ruddock, Benjamin Brown, Mei Yee Tang, Harm Van Marwijk

**Affiliations:** 1 Centre for Primary Care, Division of Population Health, School of Health Sciences, Faculty of Biology, Medicine and Health, University of Manchester, Suite 3, 6th Floor, Williamson Building, Oxford Road, Manchester, M13 9PL UK; 2 Health eResearch Centre, University of Manchester, Vaughan House, Portsmouth Street, Manchester, M13 9GB UK; 3 PRIMER, University of Manchester, 5th Floor Williamson Building, Oxford Road, Oxford, M13 9PL UK; 4 Centre for Health Informatics, Division of Informatics, Imaging & Data Sciences, The University of Manchester, Oxford Road, Manchester, M13 9PL UK; 5 Division of Primary Care and Public Health, Brighton and Sussex Medical School, University of Brighton, Room 318a Mayfield House, Falmer, Brighton, BN1 9PH UK

**Keywords:** PPI, Patient and public involvement, Public engagement, Health check, Patient records, Lifestyle, Cardiovascular risk, Funding application

## Abstract

**Background:**

Following an initial NHS Health Check appointment, the National Institute for Health and Care Excellence (NICE) suggest patients with QRISK2 scores of ≥10% should be offered advice on lifestyle and the risks and benefits of starting a statin. NICE recommend GPs should ascertain patients’ pre-existing knowledge of cardiovascular disease risk, explore health beliefs, assess readiness to change, offer support, and engage family members. Condensing this complex discussion into a short consultation may result in inadequate patient understanding of the benefits of preventive measures. An alternative approach is needed. We propose a digital adjunct giving patients the opportunity to interact with their health check results from home before returning to see their GP. Before embarking on funding applications we sought the views of patients and members of the public.

**Methods:**

We consulted the Primary Care Research in Manchester Engagement Resource (PRIMER), an established departmental Patient and Public Involvement (PPI) group (*N* = 9) and then ran a workshop with 19 members of the public, co-facilitated by 4 members of PRIMER. Following a brief presentation on the background to the project, attendees were split into four groups and introduced to Ketso, a toolkit for creative engagement. Ketso was used to encourage group discussions regarding the project idea.

**Results:**

This PPI work improved the study design and proposed intervention. Discussions focussed on three themes: 1) positive feedback, 2) challenges and solutions, and 3) improvements/alternatives. Positive feedback included benefits to the NHS and patients. Challenges identified related to: 1) access, 2) data security, 3) engagement, and 4) negative consequences. Workshop members generated various solutions to these challenges and made additional suggestions for improvement relating to: 1) population (e.g. also including those with QRISK2 scores ≤10%), 2) duration (e.g. ongoing access to provide continued feedback), and 3) platform content (e.g. signposting to relevant services).

**Conclusions:**

This PPI work helped identify potential challenges and solutions not previously considered by the research team. Findings have informed the subsequent intervention design and strengthened the bid for funding. We aim to ensure ongoing patient and public involvement in all future stages.

## Plain English summary

In England, healthy people aged 40–74 years are eligible for an NHS Health Check. This involves an appointment with a nurse or healthcare assistant who takes a blood sample, checks blood pressure, weight, and asks about lifestyle and family history of illness. Based on the results, a person’s risk of having a stroke or a heart attack in the next 10 years is calculated. Those with a risk greater than or equal to 10% are invited to a General Practitioner (GP) appointment to discuss ways to reduce their risk. We would like to improve the system by making a website where people could ‘play’ with their health check results from home before seeing their GP. This would give them time to think about their results and see how making changes (like stopping smoking or losing weight) could reduce their risk of having a stroke or heart attack. Giving people this opportunity could make the follow up GP appointment more useful. We gathered the views of patients and members of the public to help ensure that our idea is fit for purpose. We first met 9 members of the Primary Care Research in Manchester Engagement Resource (PRIMER), and then ran a workshop with 19 people to explore the idea further. The main themes were: 1) positive feedback, 2) challenges and solutions, 3) improvements/alternatives. This work helped identify challenges and solutions not previously considered by the research team. Findings have informed the design of the study and strengthened the bid for funding.

## Background

### The NHS health check

Since 2009, individuals aged 40–74, with no pre-existing health conditions, living in England have been eligible for an NHS Health Check (NHSHC), which aims to reduce their risk of cardiovascular disease (CVD) [1]. The latest evidence suggests that the NHSHC has improved detection of chronic disease and led to improvements in blood pressure, body mass index and CVD risk scores [2]. There is good evidence that the programme has increased the number of eligible individuals being prescribed a statin, and whilst the evidence for reduction in CVD morbidity and mortality is mixed, one study has shown a small but significant reduction in stroke amongst those having the NHSHC [3]. The NHSHC is operationalised as follows: Eligible individuals are invited to an initial assessment, usually with a nurse or health care assistant, who should record demographic details such as age, gender, ethnicity and postcode. At this appointment, individuals should also be asked about smoking, alcohol consumption, physical activity, and if there is a family history of cardiovascular disease. The healthcare practitioner (HCP) should also record blood pressure, body mass index (BMI: weight in kg / height in metres^2^), and take a blood sample to measure cholesterol if this is not already available [4].

Responses and results from the initial NHSHC appointment are then entered into a risk assessment tool, such as the QRISK2 tool [5] recommended by the National Institute for Health and Care Excellence (NICE) [6]. Those with a risk score of ≥10% (i.e. risk of having a stroke or heart attack in the next 10 years) should then be invited back for a GP appointment to discuss their risk score and how they might reduce this risk [6]. NICE guidelines recommend that healthcare practitioners should: find out what an individual already knows about their CVD risk, explore their health beliefs, and assess their readiness to make lifestyle changes (and confidence in doing so). The HCP should also inform them about the ways they can reduce their risk, involve them in development of a shared management plan, and check that they have understood what has been discussed [6]. In addition to this, individuals with a risk of ≥10% should receive advice regarding diet, exercise, alcohol consumption, and smoking cessation. If appropriate, individuals should be referred to weight loss, smoking cessation, or alcohol counselling services. The evidence for such lifestyle changes in CVD risk reduction is well established [7]. If lifestyle changes are not possible or are ineffective, the pros and cons of starting a cholesterol lowering medication (statin) should be discussed and individuals should be offered this option. HCPs should be supportive, engage family members where relevant, and accept that lifestyle change may require repeated efforts [6]. After their initial health check, healthy individuals should be offered an NHSHC every 5 years [4]. Whilst NICE acknowledge that it may not be feasible for all of these areas to be covered within an initial assessment and follow up appointment, we propose that the message of the NHSHC could be more effectively presented by better preparing individuals for the follow up appointment with their GP, and encourage shared decision making [8].

### Improving the NHS health check

Between 2013 and 2018 86.4% of the eligible population in England were offered an NHSHC but only 48.5% went on to receive one [9]. Enabling individuals more time to examine and interact with their NHSHC results may increase engagement and the likelihood of the programme leading to health behaviour change and subsequent positive health outcomes. When developing or improving health behaviour change interventions it is important to consider the behaviour change techniques being employed and the evidence for these techniques [10]. The behaviour change technique (BCT) taxonomy lists 93 hierarchically clustered BCTs, allowing researchers to compare the effectiveness of various techniques, explore the mechanisms by which they effect change [11], and consider which combinations of BCTs may be most effective [12]. ‘Patient activation’ refers to ‘the knowledge, skills, and confidence a person has in managing their own health care’([13], p3) and overlaps with other constructs within psychological theories of behaviour such as ‘self-efficacy’ [14] or ‘perceived behaviour control’ [15]. Increased patient activation should lead to health behaviour change, and is related to better clinical outcomes and satisfaction with healthcare services [13]. We propose that adding an extra step to the NHSHC could improve its impact by increasing patient activation using a number of BCTs described in the behaviour change taxonomy, such as: shaping knowledge, providing information about health consequences, social comparison, comparison of outcomes, and enhanced self-belief. This proposed extra step would involve access to a website which would enable individuals to interact with their own personal NHSHC results in a user friendly way. For example, they could turn a virtual dial to examine the effect that losing weight could have on their risk score, flick a virtual switch to see the effect of stopping smoking, or explore the benefits and risks of starting a statin. Such an individualised approach might help increase the ‘salience of consequences’ of not changing, another BCT from the behaviour change taxonomy.

### Patient and public involvement

Before setting up a research study it is advisable to involve patients and members of the public [16]. Patient and public involvement (PPI) in research refers to “research being carried out ‘with’ or ‘by’ members of the public rather than ‘to’, ‘about’ or ‘for them.” [17] (p6). Evidence of having conducted PPI work is often a pre-requisite in funding applications to ensure that the proposed research is acceptable, relevant, timely, and of high quality [16, 17]. Patient and public involvement should go beyond helping design the study and play a role in all stages of the research cycle from identifying and prioritising research topics, through to monitoring and evaluation [16]. The National Institute for Health Research (NIHR) note that in the early stages of a project, patients and the public may help with; clarifying and affirming the importance of the research question, ensuring the appropriateness of the methodology, and reviewing recruitment, consent, and data collection methods. Patients and the public may also help with identifying barriers to participation in a study, exploring ethical issues, and with writing the plain English summary. Conducted correctly, PPI can be highly beneficial in improving funding applications, running the research project itself, dissemination, and ensuring impact [16]. We therefore involved patients and members of the public at an early stage of our research to better understand a) how we can improve engagement of the public with the NHSHC, and b) if an interactive platform incorporating a modifiable risk score could help achieve this aim. In this paper we focus on the impact PPI has had on our research idea and subsequent proposal.

## Methods

### Initial consultation

We first consulted the Primary Care Research in Manchester Engagement Resource (PRIMER) group. PRIMER is an established Patient and Public Involvement (PPI) group of volunteers which receives support from the University of Manchester School of Health Sciences and the NIHR School for Primary Care Research. Nine PRIMER members (six female, three male) attended a 1 h consultation group on 21st June 2017 to provide feedback on the proposed research. BM presented a summary of the current NHSHC programme, followed by a description of the idea of adding an extra step to this and the next stage of PPI work (i.e. a PPI workshop). Topics for discussion included: 1) Proposed project design (including the importance of the research question, research design, and recruitment), 2) The proposed intervention, 3) Data collection methods (including the primary outcome measure), 4) Future grant development, and 5) The future PPI workshop, and 6) Any other issues the group members wished to raise. The group session was chaired by one of the PRIMER members (AR). Two members of the research team were present, one leading the discussion (BM) and the other acting as an observer (SF). All participants had been emailed a copy of the plain English summary of the project prior to the consultation, along with recruitment materials for the workshop. The main points raised were noted by the observer. After the session, BM emailed group members a summary of the discussions for information and further validation. The PRIMER members were also asked if they wished to help facilitate a PPI workshop to garner further input and feedback on the proposed project. The summary was amended based on the feedback received, and redistributed. Six members of PRIMER indicated an interest in helping facilitate the upcoming workshop, although 2 subsequently had to withdraw due to other commitments.

### Workshop planning and facilitator training

The PRIMER members who had indicated an interest in helping facilitate the workshop were invited to attend a second meeting to discuss the content and design of the workshop, and to receive training on facilitation techniques and the use of Ketso, a toolkit for creative engagement [18]. Four PRIMER members attended this 2 h meeting on 2nd August 2017. The workshop content and design was further refined during this meeting and PRIMER members were given the opportunity to practice facilitation techniques, using Ketso, and receive feedback. Refreshments were provided and PRIMER members were reimbursed for their time and expenses as per INVOLVE guidelines [17]. One PRIMER member (ML) and two researchers (BM, SF) attended a subsequent Ketso training day organized by Bury NHS Clinical Commissioning Group on 20th Sept 2017. Those who were unable to attend this additional training were sent copies of handouts from the day.

### Workshop

Workshop participants were recruited via paid Facebook advertisements, posters in the local area, and snowballing. Recruitment materials (available from the authors on request) and methods had been refined following feedback from the first PRIMER meeting to ensure a more diverse group of patients and public. We aimed to recruit 20 participants who could be split into four groups of five for each PRIMER facilitator as this was felt to be a comfortable group size for all group members to simultaneously engage with the Ketso boards. Forty-nine members of the public aged 40 years and over (i.e. more at risk of CVD and eligible for the NHSHC) and registered as a patient at a GP practice responded to the advertisements (22 male, 27 female; 12 from Facebook) and 19 were able to attend (9 male, 10 female; 4 recruited via Facebook) the workshop on 17th October 2017. Participants came from a diverse range of backgrounds and included a librarian, a cleaner, a retired fireman, a university lecturer, an author, an editor, a mature student and a speech and language therapist. Refreshments were provided and both PRIMER members and workshop attendees were reimbursed for their time and expenses as per INVOLVE guidelines [17]. Ethical approval was not required [17, 19].

The workshop began with a question: “How can we use electronic GP records in a way that would help patients be more involved with their NHS Health Check?”. Whilst attendees were considering this question, BM presented a summary of the NHS Health Check, and how it is currently operationalised. Next, BM described the idea of adding an extra step to the NHSHC involving access to an interactive website enabling people to interact with their NHSHC results before returning to see their GP. Attendees were then introduced to Ketso and the manner in which it would be used during the workshop was explained, drawing upon techniques gleaned from the previous Ketso training sessions and the Ketso website [20]. Ketso resembles a physical mind-map made from a felt board, the central topic (or trunk) is surrounded by branches (representing themes) onto which group participants can stick leaves (representing thoughts or ideas) of different colours. Using Ketso correctly ensures that everyone in the group has the chance to express their views [18] and the resulting board of ideas serves as a useful aide memoir alongside notes taken during the workshop. Different coloured leaves represent different categories of ideas. In this workshop we chose to represent positive feedback with yellow leaves, new ideas as green leaves, potential challenges as grey leaves, and potential solutions to these as green leaves.

After being introduced to Ketso, workshop attendees were split into four groups, each of which sat at a table with a PRIMER facilitator. Attendees engaged in a warm up exercise to familiarise themselves with using Ketso and to ensure they understood the principles of using it, such as sharing an idea on the board, briefly explaining it, letting others have a turn without interruption, and writing down any subsequent ideas on leaves which are then placed on an appropriate branch. After the warm-up activity, facilitators turned over the Ketso board to show a partly constructed ‘ideas tree’ (generated during discussions with the PRIMER group), the trunk being “Getting the most out of your NHS Health Check”, and topics for discussion (or branches) being: 1) who might use it, 2) what should it look like, 3) How will it fit into the current system, 4) security/confidentiality, and 5) alternative ideas. These topic areas had been decided upon during previous discussions with the PRIMER group. Workshop participants were also advised they could add extra ‘branches’ to the ‘tree’.

Tables then engaged in a number of time-limited Ketso activities, namely considering the following questions (generated during discussions with the PRIMER group): 1) “How could we improve the NHS Health Check”, 2) “What do you like about the proposed research idea?”, 3) “How could this fit in with the current NHSHC?”, 4) “What are the possible challenges”, 5) “How could we overcome these challenges?”, and 6) “What are the most important ideas on your board?”. During these exercises attendees also had the opportunity to swap tables, review another group’s Ketso board, and comment on its contents. On completion of the exercises, table members were encouraged to indicate 3 or 4 ideas which they felt were most important by placing a marker beside them, and feedback to the group as a whole. Facilitators made hand written notes of the discussions at each table, and photographs were taken of the Ketso boards to serve as an aide memoir for later data analysis. The workshop was not audio or video recorded. A report of the workshop was produced and distributed to PRIMER facilitators for information and validation. Once PRIMER feedback had been incorporated into the report it was then e-mailed to all workshop attendees for information and further validation.

## Results

### Initial PPI consultation with PRIMER

The PRIMER group gave positive feedback about the proposed research idea. They affirmed the importance of the research question and reported that the suggested methods would be appropriate from a patient perspective. The main points drawn from the discussion related to proposed intervention study itself, although some useful suggestions were also made regarding the proposed PPI workshop. Issues raised related to; recruitment, data collection, intervention design and content, funding, and the future PPI work.

#### Recruitment

PRIMER members were largely supportive of the idea of recruitment for the workshop via paid Facebook advertisements, although felt that this needed to be supplemented by posters and flyers around the local area to ensure a more representative mix. We therefore amended our recruitment plans for the workshop to include posters. With regards to recruitment into the intervention itself, some PRIMER members expressed concern that the ‘worried well’ would be disproportionately represented and suggested recruitment should occur outside of GP surgeries to tackle this issue. It was also suggested that stressing the benefits of the project to patients might also help with recruitment. As some PRIMER members were unaware of the NHS Health Check programme, it was felt that there needed to be awareness-raising activities for the programme generally through mediums such as local radio and television. It was agreed to cover these topics in the workshop.

#### Data collection

One group member noted the need to specify what our baseline measures would be in order to demonstrate an interaction effect. As a result of this we spoke to some GP surgeries to ascertain which measures are taken at the initial Health Check appointment and found that this is inconsistent. For example, although Public Health England state that a NHS Health Check must record physical activity [4], not all GP surgeries currently do this. We would therefore need to ensure complete and consistent recording of baseline measures for our future study.

#### Intervention design

Most of the discussions focussed on the design of the intervention itself. Group members raised concerns that people may be worried that the introduction of this extra step into the health check could be the beginning of a trend to reduce face to face contact for patients. There was some concern that presenting patients with a risk score via a website, without a healthcare practitioner present could cause unnecessary anxiety. Current practice involves presenting NHSHC results to patients in the follow up letters they receive, so the proposed intervention does not vary widely in this respect. It was suggested that the extra step could potentially widen health inequalities as not everyone would have access to the internet. It was agreed this topic would warrant further consideration in the workshop and future qualitative work. Proposed improvements to the intervention included the inclusion of other risk scores, such as diabetes, and ongoing access to the digital platform to enable people to monitor their progress.

#### Funding

In terms of a future funding application, several PRIMER members expressed an interest in being involved. One group member questioned why the project summary they had received in advance had not specified a larger number of potential funding sources. It was agreed that additional potential funding sources would be looked into.

#### Future PPI work

The proposed workshop was discussed and several PRIMER members expressed an interest in being involved. It was agreed that the easiest way to set a future date to discuss the workshop in more depth and provide facilitator training would be to set up an online ‘Doodle Poll’.

### Facilitator training session

Although initially planned as a session to provide PRIMER members with training in facilitation skills and the use of Ketso, the facilitator training session also turned out to be a useful PPI exercise in itself. Suggestions regarding the organization of the workshop and the topic ‘branches’ for Ketso boards were incorporated into the workshop plan. Group members were concerned the Ketso boards we planned to use were too small and so additional Ketso kits with larger boards were obtained for the workshop.

### The workshop

Workshop discussions broadly focussed around three main themes: 1) Positive feedback, 2) Challenges and solutions, and 3) Improvements/alternatives. Whilst the ideas within these themes have been grouped together and labelled by the authors, the ideas themselves were generated by workshop participants. These themes are summarised in Table 1, along with related subthemes and examples of each. Ideas that were rated as being important by each of the four workshop groups are indicated by numbered superscripts, i.e. an idea with the superscripts “2, 3” indicates that workshop groups 2 and 3 rated this idea as being important.

**Table 1:** Workshop discussion themes regarding the proposed intervention^a^

Main theme	Subtheme	Examples
Positive feedback	Benefits to the NHS	• Encourage healthy lifestyles and reduce NHS costs^2,4^ • Save GP time by ensuring patients better prepared for health check consultation
	Benefits to patients	• Improved patient engagement with health professionals and with own health^4^ • Improved communication between patients, professionals, family members and carers^1,2^ • Educational (facilitating informed decision making) • Motivational (providing individualised information and feedback facilitating goal setting)^2^ • Less confrontational (i.e. impersonal advice from a screen rather than ‘judgemental’ human) • Ability to interact with own health record • Convenience of being able to view health information from a variety of locations in own time^1,3,4^
Challenges and solutions	Access	• *Literacy barriers.* Proposed solutions: appropriate reading level, choice of different ‘difficulty’ levels, training sessions in GP practices or community centres, use of ‘community champions’. • *Language barriers.* Proposed solutions: opportunities to use website at GP surgery with interpreters, provision of information in different languages • *Computer access difficulties.* Proposed solutions: provide access in GP surgeries or in community • *Disabilities.* Proposed solutions: variable text size or ‘speak aloud’ options
	Data Security	• *Family members accessing record without consent.* Proposed solutions: requirement to register for username and password with proof of identity • *Insurance companies using data to increase premiums*. Proposed solutions: Encryption of data, legal guarantees/assurances regarding security of data
	Engagement	• *Lack of patient motivation to look at results.* Proposed solution: community champions • *Low awareness of NHSHC programme.* Proposed solutions: local and national publicity campaigns • *Technology-resistant individuals.* Proposed solutions: offer the intervention in different formats, e.g. face to face, provide clear written instructions, use community outreach programmes
	Negative consequences	• *Unreliable self-reports may reduce risk scores (*e.g. *reported exercise).* Proposed solution: link to wearables such as activity trackers • *Increasing health inequalities.* Proposed solution: gather data on characteristics of users so as to better target the intervention in future • *Increased GP workload and costs.* Although cost and time savings likely to outweigh these in longer term • *Increased patient anxiety*. Proposed solution: helpline for patients^1^ • *Patient denial or reactance against results.* Proposed solution: careful presentation of results • *Concern that website may replace face to face care.* Proposed solution: clear explanation that website would serve as an adjunct to, rather than replacing, face to face care.
Improvements/alternatives	Population	• Offer website to all patients eligible for NHSHC, rather than just those with a QRISK score ≥ 10% • Expand intervention beyond NHSHC to include risk of other conditions such as diabetes or cancer
	Duration	• Expand duration of availability of platform, i.e. offer access prior to 2nd appointment and in longer term to enable patients to view changes in risk score over time as this would enable patients to view impact of dietary changes on cholesterol levels and QRISK score in longer term^2,4^
	Platform	• Ensure mobile and tablet access also available. • Allow access via an app in addition to a website. • Enable greater interactivity – e.g. allow uploading of data from home BP monitors and activity trackers. • Incorporate signposting to services e.g. smoking cessation, weight loss, blood test explanations^3^ • Incorporate social support feature, e.g. online forums • Ensure compatibility with other platforms such as Apple Health or Google Fit. • Incorporate reminder features, e.g. to have blood tests done on a specific date

^a^Examples marked with superscripts are those that small workshop groups 1 to 4 rated as important. The numbers relate to the group that rated this idea important. Ideas with more than one number were rated as being important by more than one of the groups

#### Positive feedback

Table 1 summarises examples of benefits to both the NHS and to patients discussed by workshop participants. Groups 2 and 3 rated the potential benefit of encouraging healthy lifestyles and reducing NHS costs as important. Several of the potential benefits to patients were rated as important, with the convenience of being able to view health information from a variety of locations in one’s own time being rated as important by three of the groups. Other ideas rated as important included: improved engagement with health professionals and with one’s own health; improved communication between patients, professionals, family members and carers; and the potential for the website to motivate individuals by providing individualised information and feedback and facilitating goal setting. Some workshop participants suggested that looking at the website together with family members might give people an additional incentive to adopt a healthier lifestyle.

#### Challenges and solutions

The four main challenges identified by workshop participants related to; 1) Access, 2) Data security, 3) Engagement, and 4) Negative consequences. Detailed examples of each of these subthemes are included in Table 1. It is interesting to note that although all of these ideas were generated by workshop participants, when the small groups were asked to flag the 3 or 4 most important ideas across all the topics discussed, only the potential for increased patient anxiety and the proposed solution of a helpline for patients was flagged as being important by one of the small groups.

#### Suggested improvements and alternatives

In addition to suggesting solutions to identified challenges, workshop members also made a number of suggestions regarding how the project could be improved. As shown in Table 1, these suggestions broadly fell into 3 main subthemes: 1) Population, 2) Duration, and 3) Platform. Two of the small groups flagged the idea concerning duration as important, i.e. rather than just giving people access to the website between the 2 clinic appointments; people could be given access to the website before the first appointment, and ongoing access after the second appointment. In this way, people would be able to follow their progress and see the impact the changes they had made had on their results. For example, someone who changed their diet might be able to log in a year later and examine the impact on their cholesterol level or BMI.

As shown in Table 1, there were a number of suggested improvements to the website itself. One of these ideas was flagged as being important by one of the smaller groups; the idea of incorporating a signposting service into the platform so that users could be directed to local stop smoking or weight loss services, or a website to aid with interpretation of blood test results.

## Discussion

This paper describes how we used Patient and Public Involvement to improve the research design and funding application for a project aimed at fostering a more collaborative approach to the NHS Health Check. We first received feedback from PRIMER, an established PPI group, and then went on to run a workshop with members of the public using Ketso, a toolkit for creative engagement. Our initial PPI meeting informed the subsequent workshop design, which was co-facilitated by four PRIMER members. The workshop provided valuable insights which improved the study design and proposed intervention. Discussions focussed around three themes: 1) positive feedback, 2) challenges and solutions, and 3) improvements / alternatives. Findings from the PRIMER meeting and workshop have informed and improved a subsequent funding application. We appreciate that the proposed intervention will require more than hypothetical discussions before it is operationalised, and so the funding application now incorporates proposals for further qualitative work, co-production of the intervention itself, a ‘think aloud’ study, prototype testing, and a feasibility study. Patient and Public Involvement is still a relatively new concept and is sometimes confused with participation in research [16]. Following the GRIPP2 (Guidance for Reporting Involvement of Patients and Public, 2) guidelines [21], this paper highlights the utility of PPI in ensuring that research is patient centred. We have demonstrated how PPI can uncover research design flaws that may not have been previously considered by the research team, leading to improvements in the methodology and proposed intervention. In line with similar studies [22], we found this valuable input has helped improve our funding application and feel it will ensure subsequent research engages those individuals it wishes to reach and improves its impact.

Although this PPI work has affirmed the importance of the research topic and was supportive of our proposed approach, it has changed our funding application in a number of important ways. For example, we had not previously considered that the website could be a useful communication aid between patients and family members or carers. This links with the ‘social support’ grouping of the BCT taxonomy [11]and has now been built into the proposal. Concerns regarding access have encouraged us to ensure that the website will be easy to understand, incorporating a range of presentation formats. In a similar vein, we have also addressed the issue of computer literacy by incorporating ‘community champions’ into the funding bid. We hope these amendments will help ensure we can recruit individuals with differing levels of need and ultimately encourage greater uptake of the NHSHC from a broader cross section of individuals. We were already aware of data security concerns, but have amended the bid to make it clear that participants will receive assurances that their data will not be passed on to third parties. We will also ensure that we follow the Royal College of General Practitioner guidelines on this topic [23, 24].

Two substantial changes resulting from this PPI work relate to the target population and the duration of the intervention. Our initial plan has been to target those with a QRISK2 score of ≥10% between the two Health Check appointments. Workshop members suggested that it might be better to give all participants in the intervention group access to the website, and that this access should be ongoing to facilitate self-monitoring over time (linking to the ‘feedback and monitoring grouping on the BCT taxonomy^10^). Both these changes have now been incorporated into the bid. We have also incorporated the suggestion that the website should be mobile-friendly and provide signposting to relevant services such as smoking cessation, weight loss, and exercise groups. Such signposting could facilitate behaviour change via several groupings included in the BCT taxonomy such as ‘shaping knowledge’ (e.g. information on how to perform the behaviour), ‘natural consequences’ (e.g. information about health consequences), and ‘regulation’ (e.g. pharmacological support).

Not all of the concerns raised by the PPI group could be addressed in the manner suggested. For example we felt that the suggestion of providing a helpline for those who were anxious about the results from their NHS Health Check would add an unnecessary expense to the project, but adapted this suggestion so that participants could leave a secure voicemail with their concerns for a medically qualified member of the research team to respond. The costs associated with ensuring compatibility with wearable devices or other health platforms we also felt to be too prohibitive and so have not been incorporated into the proposal at this stage.

### Limitations

When considering the findings from this work, it is important to bear in mind a number of limitations. Firstly, it is generally acknowledged that PPI activities often attract a self-selecting group unlikely to be representative of the population as a whole [22]. It is also worth noting that the NIHR INVOLVE briefing states “Researchers often ask how they can ensure that the people they involve are ‘representative’. However it is more helpful to think about seeking people’s perspectives rather than looking for people who are representative” ([16], p18). While the established PRIMER group consists of individuals with an interest in research, we attempted to obtain a more diverse sample by advertising the workshop through Facebook and posters around the local area. Workshop members were reimbursed for their time which may have decreased the likelihood that they took part solely due to their interest in the topic, indeed several attendees mentioned they had never engaged in such an activity in the past. A second potential limitation is the manner in which we used Ketso. Although Ketso training suggests the use of pre-populated branches to stimulate group discussion, providing such a priori themes is likely to have influenced the direction of discussions within in the workshop. The labels for the pre-populated branches were decided upon following discussions with the PRIMER group and so were not entirely researcher-driven. Workshop members were also provided with ‘blank’ branches around which they could cluster their own themes, and were informed they could change the branches we had supplied if they so wished. It is important to note that PPI work is different from qualitative work in that there were specific areas which we wished to discuss, and so we feel the use of a priori themes was appropriate in this case. A third potential limitation of the study is that the workshop discussions were not video or audio-recorded. Facilitators did however keep detailed notes during the table discussions, and workshop members were encouraged to write salient points on the Ketso leaves so that no important pieces of information were missed. Table facilitators were also provided with an opportunity to validate the summary of table discussions in an effort to ensure no important information was omitted. Although audio or video recording the discussions may have provided more detailed data, we felt that it would have been technically difficult due to many simultaneous conversations occurring, and that it may have made group members more reluctant to share their thoughts and ideas.

## Conclusions

This PPI work has provided many valuable insights and proved highly beneficial in improving the design and intervention of the proposed study. Feedback from both PRIMER and the workshop has helped affirm the importance of the research topic and will ensure that the intervention is more patient centred. We hope the potential impact of our proposed project aimed at increasing engagement with the NHS health check on both patients (e.g. lower CVD morbidity and mortality) and the NHS (e.g. cost savings) will be enhanced by engaging patients and the public from the beginning. Several PRIMER and workshop members have expressed an interest in continuing to help us with the next stage of the project and we intend to continue to incorporate PPI into our research. We feel other researchers should be encouraged to involve patients and members of the public from an early stage to ensure that future studies benefit from such engagement.

## Abbreviations

BCT: Behaviour change technique
BMI: Body mass index
CaVIAR: Better care via improved access to records
CVD: Cardiovascular disease
GP: General practitioner
GRIPP2: Guidance for reporting involvement of patients and public, 2
HCP: Health care practitioner
NHS: National health service
NHSHC: NHS health check
NICE: National institute for health and care excellence
NIHR: National institute for health research
PPI: Patient and public involvement
PRIMER: Primary care research in Manchester engagement resource
